# Giant epidermal inclusion cyst of the thyroid: a rare occurrence

**DOI:** 10.4322/acr.2021.318

**Published:** 2021-09-03

**Authors:** Swagatika Samal, Mukund Sable, Pradeep Pradhan

**Affiliations:** 1 All India institute of Medical Sciences, Department of Pathology and Lab Medicine, Bhubaneswar, Odisha, India; 2 All India institute of Medical Sciences, Department of ENT and Head and Neck Surgery, Bhubaneswar, Odisha, India

**Keywords:** Epidermal Cyst, Rare Diseases, Thyroid Gland, Thyroidectomy

## Abstract

Epidermal inclusion cyst (EIC) of the thyroid is extremely rare in the clinical practice. A handful of cases have been documented in the past in the world literature. A giant EIC of the thyroid is hitherto unreported. This lesion may arise from the squamous metaplasia of the thyroid follicular cells. Though non-neoplastic, giant forms can cause compression of the vital structures of the neck. In the present case, we have described a giant epidermal inclusion cyst successfully managed with surgical management.

## INTRODUCTION

EIC of the thyroid is extremely rare in the clinical practice, which is thought to be derived from the foci of squamous metaplasia^1^. Although various theories have been proposed in the past for its origin, the exact cause is still not clear.

EIC usually results due to the ingrowth of the epidermis into the dermis which could be due to a physical insult or surgical trauma. They can also be detected rarely in patients with Gardner's syndrome on the head and neck.^2^ A handful of cases have been documented in world literature.^3^^-^16 Patients usually present with a painless neck swelling and the majority of them present with a thyroid nodule. Dyspnea or dysphagia is very rare unless the swelling is large enough to compress the adjacent structures. In the present case, we have reported a giant EIC, with special concern to the cytological and histopathological characteristics after successful excision of the lesion.

## CASE REPORT

**Figure 1:**
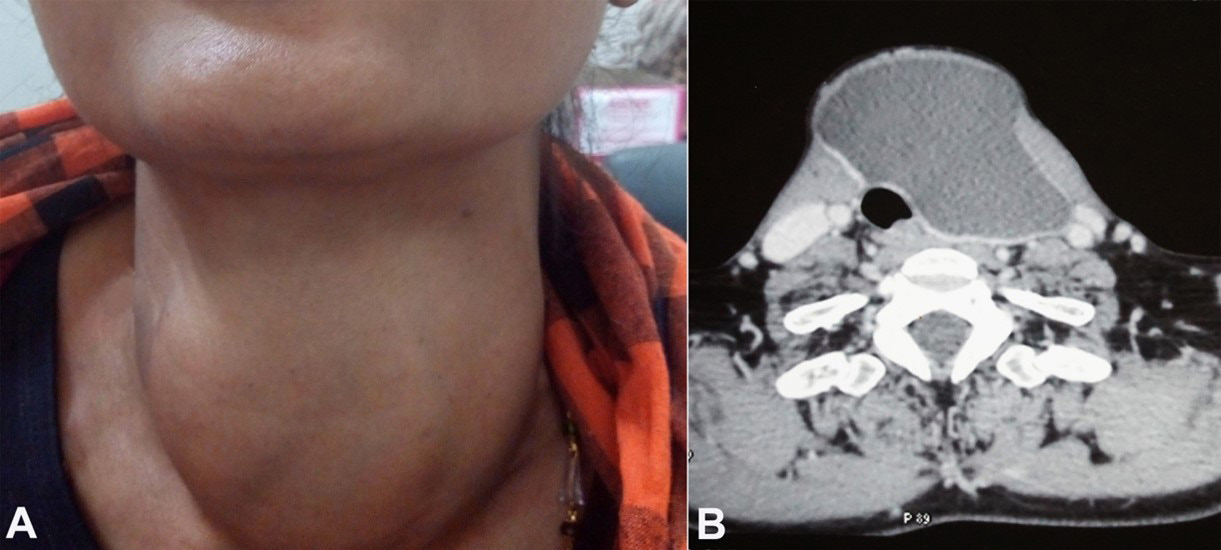
**A** – Gross view of the diffuse thyroid swelling; **B** – Contrast enhanced CT scan of the neck showing a large hypodense cystic lesion involving the left lobe of thyroid and partly the isthmus.

A 46-year-old female patient presented with a slow-growing swelling over the left side of the anterior neck for 12 years and dysphagia over the last two months. On the physical examination, a soft cystic thyroid mass (left side) was detected, which was approximately 10x10 cm in size (Figure 1A). The mass moved with deglutition but not with the protrusion of the tongue. Thyroid functions tests were normal. The ultrasonography revealed a large cyst in the thyroid, measuring 9x8x8 cm in the greatest dimensions involving the left side of the anterior neck, compressing and almost replacing the entire thyroid left lobe. A contrast-enhanced CT scan of the neck revealed a large hypodense cystic lesion involving the left lobe of the thyroid along with the isthmus, and an intact right lobe (Figure 1B).

**Figure 2:**
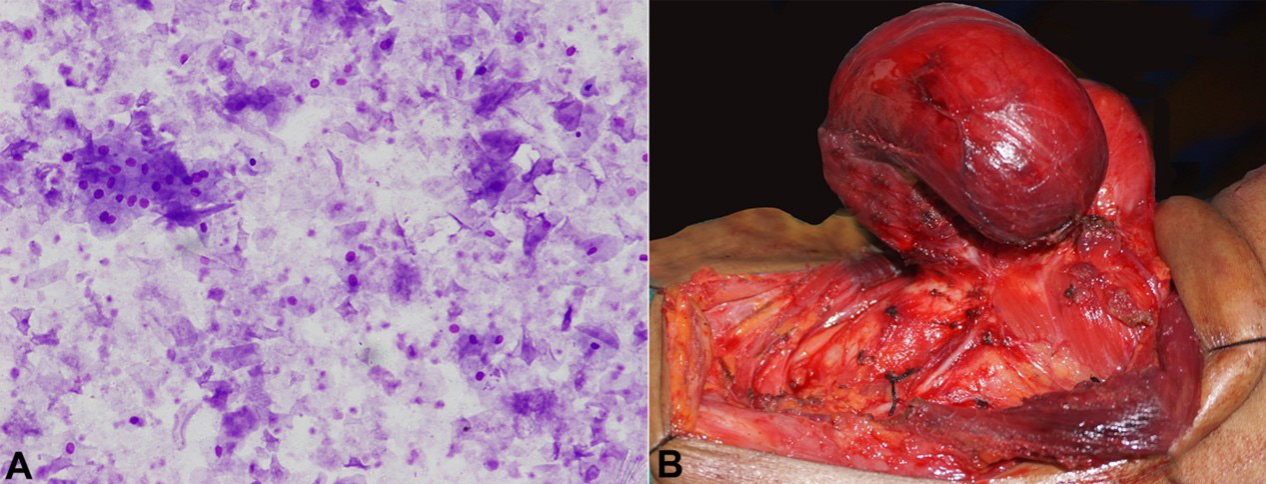
**A** – Fine needle aspiration smear showing sheets of anucleated squames admixed with few nucleated squamous cells without any nuclear atypia (Leishman stain; 200x); **B** – Intraoperative photograph showing complete removal of the tumor with preservation of the neurovascular structures.

Repeated fine needle aspiration was performed, and approximately 30 ml of dirty brown colored, foul-smelling fluid was drained, without a significant reduction in the size. The cytological smear showed sheets of anucleated squames admixed with few nucleated squamous cells (Figure 2A). No cellular atypia, necrosis, granuloma, thyroid epithelial cells, or colloid were detected. The patient had undergone a left hemithyroidectomy with complete preservation of the ipsilateral recurrent laryngeal nerve and the parathyroid glands (Figure 2B).

**Figure 3:**
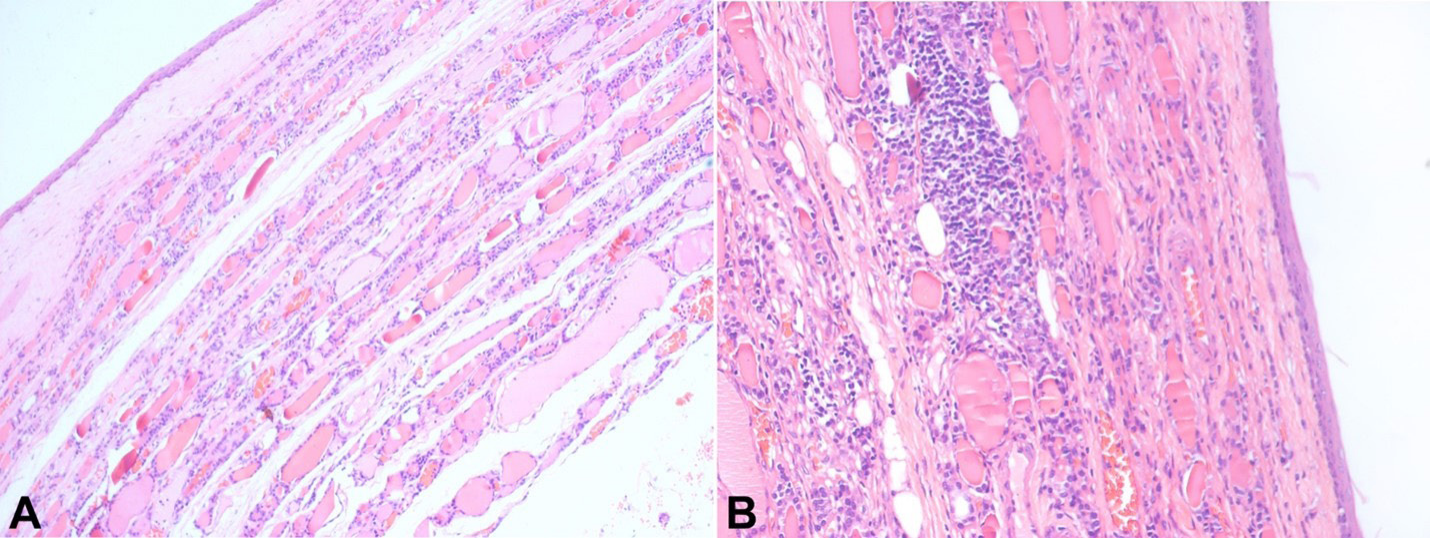
**A** – Photomicrograph of the surgical specimen showing a cyst wall lined by benign squamous epithelium with rim of compressed benign thyroid parenchyma (H&E; 100x); **B** – Focally the cyst wall shows moderate infiltration of lymphocytes with formation of lymphoid aggregate in the adjacent surrounding thyroid parenchyma (H&E; 200x).

On macroscopic examination, the left hemithyroidectomy specimen weighed 80 g. The thyroid capsule was intact with a tense cystic swelling involving the left lobe of the thyroid. On the cut section, a well-defined and unilocular cyst without a solid component was detected measuring 11.0 x 6.0 x 4.5 cm replacing the entire left lobe of the thyroid gland**.** The cavity of the cyst was found to be filled with brownish-colored, foul-smelling fluid. The cystic wall thickness measured between 0.1 and 0.2 cm. There was a thin rim of compressed thyroid parenchyma surrounding the cyst wall. On microscopic examination, the cyst was lined by stratified squamous epithelium, surrounded by compressed benign thyroid parenchyma (Figure 3A). The surrounding thyroid parenchyma shows foci of chronic inflammatory infiltrate with lymphoid aggregates (Figure 3B). There was an absence of any ciliated columnar epithelium or any other mesenchymal component.

**Figure 4:**
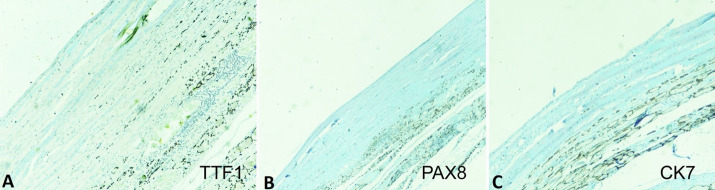
Photographs showing the negative reaction for **A** – TTF1; **B** – PAX8; **C** – CK7.

Keeping in mind the most common differential diagnosis being the thyroglossal cyst, immunohistochemical markers (TTF1, PAX8 and CK7) were undertaken. The histopathological sample was found negative for all the mentioned markers specific for thyroglossal cyst (Figure 4).

**Figure 5:**
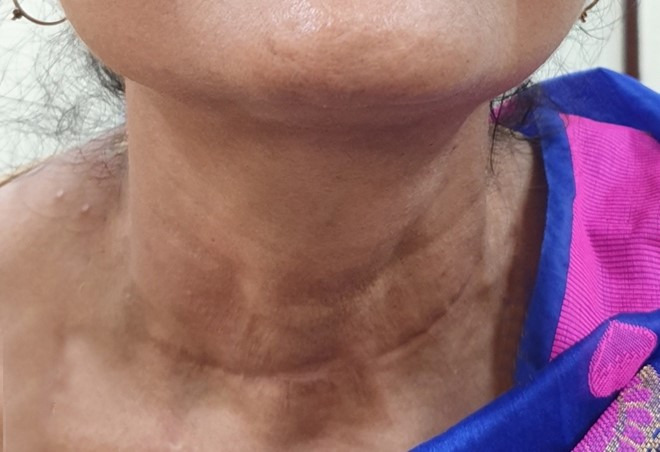
Follow up photograph (3 months) with healed scar and without evidences of recurrence.

With the above clinical-histomorphological finding, a final diagnosis of the thyroid gland’s EIC was made. The patient is doing well and is on regular follow-up for the last 18 months in the thyroid clinic without any clinical and radiological evidence of recurrence of the disease (Figure 5).

## DISCUSSION

EIC of the thyroid is rare, and only sixteen cases have been reported worldwide (Table 1).

**Table 1:** A comparative representation of the reported cases of EIC thyroid

Authors	Age(y)/Sex	Presentation	Duration	Location	size	Cavity	TFT	CD	FNAC	Surgery	HP	Follow up
Impieri^2^	26/F	NS	NA	L	NA	UL	NA	NA	NA	Excision	EIC	NA
Sonnino^3^	4/F	NS	NA	R	2.0	UL	NA	NA	NA	Lobectomy, Sistrunk	EIC	NA
Sonnino^3^	9/F	NS	NA	L	3.0	UL	NA	NA	NA	Excision	EIC	NA
North^4^	78/M	NS	NA	I	2.0	UL	NA	NA	NSC	HT	EIC	NA
North^4^	58/M	NS	NA	R	1.0	UL	NA	NA	NSC	HT	EIC	NA
Hatada^5^	50/F	NS	NA	R	4.4	UL	NA	NA	NSC	Lobectomy	EIC	NA
Salib^6^	60/M	D	3Y	L	3.0	UL	ET	Malignancy	NA	Lobectomy	EIC	NR at 6M
		NS	2W									
Chen^7^	54/M	NS	Incidental	R	3.2	UL			NSC	Lobectomy	EIC	NA
Bekele^8^	27/M	NS	10Y	L	3.2	UL	ET	NA	Inadequate	HT	EIC	NA
Palombini^9^	38/F	**	NA	L	1.0	UL	NC	NA	NSC, ANSC	HT	EIC	NA
Rumberger^10^	71/F	NS *	NA	R	1.3	UL	HpT	NA	NSC	Lobectomy	EIC	NA
Kuduban^11^	28/M	NS	2Y		NA	NA	NA	NA	NA	Cystectomy	EIC	NR at 12M
Choure 12	46/F	NS	6M	R	3.0	UL	ET	TC		HT	EIC	NR
Kulkarni^13^	26/F	NS	6M	NA	3.0	UL	ET	NG	NSC,ANSC	HT	EIC	NA
Binayke^14^	26/F	NS	6-7 M	R	1.5	UL	ET	NCG	NSC,ANSC, Inflammatory cells	HT	EIC FBGC	NA
Mansi^15^	28/M	NS	10Y	R	6.0	UL	ET	CG	NSC,ANSC	HT	EIC	NR
Our case	46/F	NS	12Y	L	11.0	UL	ET	CC	NSC, ANSC	HT	EIC	NR at 18M
		Dpg										

CC= Colloid cyst; CG= Colloid goiter; D= dysphonia; Dpg= Dysphagia; EIC= Epidermal inclusion cyst; ET= Euthyroid; F= Female; FBGC= Foreign body giant cell reaction; HT= Hemithyroidectomy; HpT= Hypothyroid; I= Isthmus; L= Left lobe; M= Male; M= Months; NA= Not available; NC= non-contributory, NCG= Nodular colloid goiter; NR= No recurrence; NS= Neck swelling; R= Right lobe; TFT= Thyroid function test; UL= Unilocular; Y=Year.

* Gurgling sensation.

** Non palpable cold thyroid nodule.

The literature search was undertaken in PubMed, Google scholar, and Scopus database up to June 4, 2020. Our search was performed using the Keywords as follows: Inclusion cyst AND Thyroid. Although the squamous epithelium is not a part of the normal thyroid gland, it may be detected in various lesions, including the metaplasia, neoplasm, and congenital remnants.^17^ Thyroid follicular cells can undergo squamous metaplasia, particularly in a background of long-standing colloid goiter or chronic inflammation, as in Hashimoto thyroiditis.^18^^,^^19^ In the present case, the absence of colloid in aspiration and presence of a well-defined large cyst entirely lined by squamous epithelium excludes the possibility of a colloid goiter. Also, the presence of foci of dense lymphocytic infiltration with the formation of lymphoid aggregates in the adjacent surrounding thyroid tissue raised the suspicion of an inflammatory etiology. However, the long-standing history, and the lack of evidence of a functional abnormality pointed towards the inflammation as a secondary event in a long-standing keratinous cyst. The neoplasms like papillary thyroid carcinoma, follicular thyroid carcinoma, and anaplastic thyroid carcinoma show squamous differentiation. A few cases demonstrate the origin of squamous cell carcinoma from the above primary thyroid malignancies has been reported. In the index case, the clinical finding, cytologically bland-looking cells without any atypia or any associated atypical follicular lesion, excluded the possibility of any neoplastic etiology. Among the congenital anomalies, remnants of the thyroglossal duct, branchial cyst, and dermoid cyst are documented to show the presence of squamous cells.^17^ A few authors considered EIC of the thyroid as an intraparenchymal thyroglossal duct cyst. Nevertheless, the thyroglossal cyst has a typical midline location with a columnar cell, a fibrous cyst wall with associated inflammation. The squamous cells of the thyroglossal cyst typically resemble squamous metaplasia of the cervical smear.^20^ Intra thyroidal branchial cleft remnants have been reported. However, in the present case, the absence of lymphoid infiltrate with a germinal center in the submucosa can easily exclude this possibility.^21^ The absence of any adnexal structures or any mesenchymal derivatives in the cyst wall excludes a dermoid cyst’s possibility in the present case. Table 1 summarizes the clinicopathological parameters of the thyroid EIC reported cases.^3^^-^16

The age of the patients ranged from 4 to 78 years. Females are most commonly affected. The tumor size varied from 1.0 to 6.0 cm in dimension, in contrast to the present case, which was 11.0 cm in its longest axis, a giant cyst almost replacing the entire left lobe of the thyroid displacing the contralateral lobe**.** The cytology showed sheets of anucleated squamous cells mixed with few nucleated squamous cells with the absence of cellular atypia, granuloma, or thyroid epithelial cells, as described in the literature. Some authors reported the presence of inflammatory cells in addition to the benign squamous cells.^15^ The presence of benign squamous cells without any atypia is considered benign as per the Bethesda system of reporting thyroid aspirates.^22^ On gross examination, the epidermal inclusion cyst is unilocular, well-circumscribed, containing dirty brown material. They were microscopically lined by the stratified squamous epithelium, a similar picture obtained in the present case. Again the immunohistochemistry was negative for all the markers (TTF1, PAX8 and CK7), specific for thyroglossal duct cyst. Due to the lesion’s benign nature, most of the patients are treated with surgical excision of the cyst, lobectomy, or hemithyroidectomy, as demonstrated in the present case, where the patient had undergone hemithyroidectomy.

## CONCLUSION

EIC of the thyroid is extremely rare in clinical practice. A giant cyst of the thyroid almost replacing the entire hemi thyroid is yet to be described in the literature. Although fine-needle aspiration is considered an important investigation for the preoperative diagnosis, it is still not always confirmatory. The possibility of an EIC should be kept in mind while finding benign squamous cells without any follicular cells in a thyroid aspirate and can be managed successfully with hemithyroidectomy.

## References

[1] Harcourt-Webster JN (1966). Squamous epithelium in the human thyroid gland. J Clin Pathol.

[2] Pandya KA, Radke F (2009). Benign skin lesions: lipomas, epidermal inclusion cysts, muscle and nerve biopsies. Surg Clin North Am.

[3] Impieri M, Russo R, Cappagli M, Cucchi I, Poggi P, Calcina GG (1987). Epidermoid cyst of the thyroid: report of a case. Acta Chir Belg.

[4] North JH, Foley CAM, Hamill LRL (1998). Intrathyroid cysts of thyroglossal duct origin. Am Surg.

[5] Sonnino RE, Spigland N, Laberge JM, Desjardins J, Guttman FM (1989). Unusual patterns of congenital neck masses in children. J Pediatr Surg.

[6] Hatada T, Ichii S, Sagayama K (2000). Intrathyroid thyroglossal duct cyst simulating a thyroid nodule. Tumori.

[7] Salib RJ, Radcliffe G, Gallimore A (2001). Intra-parenchymal thyroid epidermoid cyst presenting with a left recurrent laryngeal nerve palsy. J Laryngol Otol.

[8] Chen KTK (2007). Fine-needle aspiration cytology of epidermoid cyst of the thyroid: report of a case and review of seven cases. Diagn Cytopathol.

[9] Bekele Y, Gerscovich EO, Naderi S, Bishop J, Gandour-Edwards RF, McGahan JP (2012). Sonography of an epidermoid inclusion cyst of the thyroid gland. J Ultrasound Med.

[10] Palombini L, Cozzolino I, Luigi S, Pifano A (2015). Fine needle cytology of intrathyroid epidermoid cyst. Diagn Cytopathol.

[11] Rumberger LK, Mancini M, Curzon M, Orucevic A (2014). Intrathyroidal epidermoid cyst associated with nodular Hashimoto’s thyroiditis: a rare pathologic finding in a common clinical entity. Am Surg.

[12] Kuduban O (2015). Epidermal Inclusion cyst of thyroid gland. Eurasian J Med.

[13] Choure DD, Nichat PD, Agarwal S, Turabi MA, Tayade MB (2015). epidermal cyst presenting as a solitary thyroid nodule: a rare case report. J Clin Med Res.

[14] Kulkarni SS, Vyas AS (2016). Thyroid epidermal cyst – a common cyst, rare site. NJMR.

[15] Binayke R, Deshpande KP (2017). Epidermal inclusion cyst of the thyroid gland: an uncommon entity. Int J Curr Res Med..

[16] Mansi JP, Riti TKS (2017). A rare case of epidermal inclusion cyst of thyroid gland. J Med Sci Health.

[17] LiVolsi VA, Merino MJ (1978). Squamous cells in human thyroid gland. Am J Surg Pathol.

[18] Nayak SK, Pai PK, Naik R, Rao VS (2002). Extensive squamous metaplasia in nodular goiter: a diagnostic dilemma in the fine needle aspiration (FNA) cytology: a case report. Indian J Pathol Microbiol.

[19] Kobayashi T, Okamoto S, Maruyama H, Okamura J, Takai S-I, Mori T (1989). Squamous metaplasia with Hashimoto’s thyroiditis presenting as a thyroid nodule. J Surg Oncol.

[20] Shaffer MM, Oertel YC, Oertel JE (1996). Thyroglossal duct cysts. Diagnostic criteria by fine-needle aspiration. Arch Pathol Lab Med.

[21] Louis DN, Vickery AL, Rosai J, Wang CA (1989). Multiple branchial cleft-like cysts in Hashimoto’s thryroiditis. Am J Surg Pathol.

[22] Cibas ES, Ali SZ (2009). The bethesda system for reporting thyroid cytopathology. Am J Clin Pathol.

